# Response of soil fungal community structure and co-occurrence network features on plantations of limestone mountains along habitat specialization gradient

**DOI:** 10.3389/fmicb.2025.1691167

**Published:** 2025-11-03

**Authors:** Zhenlu Qiu, Jin Liu, Ting Pan, Ziwen Wang, Yanru Shen, Xueting Wang, Jing Shu

**Affiliations:** College of Forestry Engineering, Shandong Agriculture and Engineering University, Jinan, Shandong, China

**Keywords:** habitat-generalists, habitat-specialists, network, afforestation, soil fungi

## Abstract

**Introduction:**

This study elucidated how different plantation types in limestone mountains shape the community structure and co-occurrence networks of soil fungal habitatspecialization groups, offering a habitat-adaptation perspective on the assembly mechanisms of soil microbial diversity during ecological restoration.

**Methods:**

In this study, we grouped soil fungi from plantations of coniferous forests (CF), mixed forests (MF) and broad-leaved forests (BF) into habitat-generalists, specialists and opportunists based on niche breadth, and examined how forest type shapes their diversity, community structure and co-occurrence networks along a habitat-specialization gradient.

**Results:**

We found that: (1) The number and abundance of habitat-specialists significantly exceeded those of generalists. Habitat-generalists exhibited the highest abundance and ecological niche width in BF, whereas habitat-specialists were most abundant in CF. (2) The diversity index was the highest in BF across all habitat specialization groups. Differences in community structure among forest types increased with habitat specialization, and the composition of dominant tree species significantly influenced the community structure of each group. Soil properties primarily affected the community structure of habitat generalists and opportunists between BF and other forest types. (3) The network structure of habitat specialists exhibited high modularity, while habitat generalists formed independent subnetworks with more fragile structures. CF and BF exhibited strong intra-module connections and high modularity, whereas MF displayed high intermodule connectivity, which reduced their modularity. Both within-module (Zi) and between-module connectivity (Pi) of increased with habitat specialization and the proportion of broadleaved tree species.

**Discussion:**

We concluded that broad-leaved plantations, by increasing soil environmental heterogeneity, promoted the diversity of habitat-specialists and enhancing their network hub roles, representing the optimal strategy for optimizing below-ground biodiversity and stability in limestone mountain forest restoration.

## 1 Introduction

The biological and abiotic components in forest soil interact with various environmental factors, interconnecting through material cycling and energy flow, thereby facilitating their turnover and stability, and forming a soil ecosystem (Zhu et al., 2022). Soil microorganisms, as the most important biological components of soil ecosystems, typically exhibited rapid responses and high turnover rates to environmental changes. The ecological linkages between aboveground and belowground biota during forest succession have been considered an important mechanism in forest development and succession (Guo et al., 2024). For example, changes in plant species strongly influenced the community structure and function of soil microorganisms (Nagati et al., 2018). The key factor is that litter input alters soil abiotic properties such as pH and nutrient availability (Deng et al., 2016). Soil fungi, acting as decomposers, symbionts, and pathogens, are essential functional components of the soil. Extracellular enzymes produced by fungi participate in the cycling and metabolism of soil C, N, and P, significantly affecting the decomposition of plant litter (Lin et al., 2018; Li et al., 2018). Fungal biomass is sensitive to changes in soil properties, and generally higher in broad-leaved forests than in coniferous forests (Noll et al., 2016). Saprophytic soil fungi can decompose soil substrates and enhance soil nutrient cycling (Nagati et al., 2018; Liu et al., 2018), while ectomycorrhizal fungi (Preetisri et al., 2017), as symbionts, are known to improve plant nutrition status (Becquer et al., 2018; Clasen et al., 2018).

Community assembly refers to the mechanisms of its diversity formation and maintenance and mainly comprises deterministic processes and stochastic processes. The former involves environmental filtering and biological interactions, while the latter includes dispersal limitation and random variation. According to Peng et al. (2021), it is a necessary factor for coupling microbial community structure with ecosystem function (Wan et al., 2021). The construction of soil fungal communities has been proved to be influenced by complex interactions between plant community changes and various environmental factors (Canini et al., 2019; Geng et al., 2023). Vegetation type significantly affected soil fungal community structures (Porazinska et al., 2018), which might be achieved by altering the chemical composition and content of litter and root exudates or by mediating through the regulation of soil physical and chemical properties (Yang and Zhu, 2015; Ortiz et al., 2022). Studies have shown significant differences in soil fungal community structures between coniferous forests and broadleaved forests (Vuong et al., 2020). Based on ecological niche width and distribution range (Abdullah Al et al., 2022), soil fungal species exhibit varying degrees of adaptability to environmental changes, with the community including habitat generalists that can adapt to substantial changes in environmental quality and habitat-specialists that can only survive in relatively narrow habitat ranges (Sriswasdi et al., 2017). Previous studies have indicated that habitat generalists and habitat specialists among soil fungi adapt to environmental changes differently, suggesting variations in their community assembly mechanisms (Zheng et al., 2023; Mo et al., 2021; Yan et al., 2022; Zuo et al., 2023). However, how different plantation types filter soil fungal communities along a habitat-specialization gradient in limestone forest remains poorly understood.

Intraspecific and interspecific interactions of soil fungi were key drivers in community assembly. Co-occurrence network analysis effectively quantified interactions by identifying potential associations between species (Faust and Raes, 2012). Network topology parameters (such as average degree, closeness centrality) can identify the key species that have the greatest impact on community stability (Banerjee et al., 2018; Li et al., 2020). Furthermore, the modular structure of complex co-occurrence networks is crucial for maintaining overall community stability and functional differentiation (Xue et al., 2018; Liu et al., 2019; Ratzke et al., 2020). Worth noting that, recent studies indicated that habitat specialists contribute more significantly to maintaining network structure than habitat generalists. (Abdullah Al et al., 2022; Yan et al., 2022).

Shandong Province is located in the North Temperate Zone, where agriculture and forestry were originally suitable for habitation. Historical deforestation has led to the degradation of most mountainous regions into barren rock (Wu, 2017; Tan et al., 2020), Severely damaged the original diversity and service functions of the forest ecosystem (Wang et al., 2025). It was not until the 1960s that fast-growing and drought-resistant tree species such as *Pinus tabulaeformis*, *P. thunbergii*, and *Robinia pseudoacacia* were intentionally introduced on thin limestone slopes, gradually establishing the existing artificial forests (Du et al., 2020). Our study area, the Fanggan Ecological Restoration Area, is located in the northern foothills of the Taishan Mountain Range in Shandong Province. Since 1970, villagers have planted over three million trees for ecological restoration, forming diverse plantation types including coniferous, broad-leaved, and mixed forests. Currently, the ecological restoration area evolved into a thriving community with a well-defined vertical structure and dense canopy, supporting the healthy growth of various forest types (Qiu et al., 2022). Consistent soil conditions before planting, due to soil transfer for vegetation recovery on barren mountains, enable a more accurate assessment of the impact of different plant types on the soil ecosystem. Previous reports have detailed the effects of plantation types on soil properties, bacterial community structure and diversity (Qiu et al., 2022) and analyzed the characteristics and mechanisms of bacterial community structure changes in response to plantation types from both habitat specialization and abundance dimensions (Qiu et al., 2023). However, studies on how the community structure and co-occurrence networks of soil fungi respond to different afforestation types along a habitat specialization gradient were still particularly lacking.

This study focused on soil fungi in coniferous forests, mixed forests, and broad-leaved forests developed in the Fanggan Ecological Restoration Area. We explored the characteristics and environmental driving mechanisms of diversity and structure responses of different habitat specialization groups to different afforest types, and conducted analyses of fungal co-occurrence network features, topological parameters, and key groups. Our research aimed to reveal: (1) differences in the response of abundance, ecological niche width, diversity, and community structure of different habitat specialization groups to afforestation types; (2) differences in the community assembly characteristics of different habitat specialization groups among afforestation types; (3) differences in network structure characteristics and key groups of different habitat specialization groups among afforestation types.

## 2 Materials and methods

### 2.1 Study site and plot sets

The Fanggan Ecological Restoration Area is located in the northern region of the Shandong Central Mountain Range, with geographical coordinates spanning from 117°24′45″ to 117°28′5″E and 36°24′23″ to 36°26′44″N. This area is characterized by mountainous and hilly terrain, reaching a maximum altitude of 860 m and featuring over 30 peaks exceeding 400 m in height. The climate of restoration zone is classified as a continental monsoon climate, with the average annual temperature at 12.4 °C, and the lowest extreme temperature record of −22.5 °C. The average value of annual precipitation exceeded 830 mm, predominantly occurred from July to September. The predominant soil type in the region is mountain brown soil, with an average litter layer of 4 cm. The vegetation community in the area displays a distinct vertical structure, but species diversity is low. The tree layer primarily comprises *Pinus densiflora* Siebold & Zucc., *P*. *tabulaeformis* Carrière, *Populus davidiana* Dode, *Robinia pseudoacacia* L., and *Diospyros lotus* L. The shrub layer is mainly composed of *Vitex negundo* var. *heterophylla* (Franch.) Rehd., *Ziziphus jujuba* var. *spinosa* (Bunge) Hu ex H. F. Chow., *Grewia biloba* G. Don, along with seedlings regenerating from arbor layer. The herbaceous layer predominantly consists of species from the Gramineae, Cyperaceae, and Compositae. All plantations within the restoration area were established between 1975 and 1985, resulting in a relatively uniform forest age.

Based on a review of relevant literature and field surveys, the forests in the restoration area are primarily located between 300 and 750 m in altitude. Consequently, an investigation into the vegetation communities was conducted in regions with dense forest canopies within this altitude range. 24 sample plots with each area of 10 m × 10 m were established, covering three plantation forest types: six coniferous forest (CF) plots, six mixed coniferous and broad-leaved forest (MF) plots, and twelve broad-leaved forest (BF) plots. To minimize the impact of topographical factors, the design of each plot aimed to have as similar topographical conditions as possible. Additionally, the geographical coordinates, elevation, aspect, and slope of each plot were recorded.

### 2.2 Vegetation community survey and soil physicochemical property analysis

Measurements were taken for each tree with DBH ≥ 3 cm. *α*-diversity indices of the vegetation community, including the Shannon-Wiener index (Equation 1) (Shannon and Weaver, 1949), Simpson diversity index (Equation 2) (Simpson, 1949), Shannon evenness index (Equation 3), Simpson evenness index (Equation 4), Pielou evenness index (Equation 5) (Pielou, 2011), and species richness were calculated.


(1) $$\text{Shannon}-\text{Winner diversity index}=-\sum_{i=1}^{m}P_{i}{\mathrm{lnP}}_{i}$$

(2) $$\text{Simpson diversity index}=1-\sum_{i=1}^{m}\frac{P_{i}(P_{i}-1)}{N(N-1)}$$

(3) $$\text{Shannon evenness index}=\frac{\text{Shannon}-\text{winner diversity index}}{\log(m)}$$

(4) $$\text{Simpson evenness index}=\frac{\text{Simpson diversity index}}{\log(m)}$$

(5) $$\text{Pielou evenness index}=\frac{-\sum_{i=1}^{m}P_{i}\log P_{i}}{\log(m)}$$

In each plot, topsoil samples within the 0–10 cm range were collected using the five-point sampling method. The sampling points were arranged at the center and four corners (3 m from the vertex of the plot). Then the samples at the five points in each plot were thoroughly mixed and then divided into two parts. As a result, there were a total of 48 soil samples. One part was stored in liquid nitrogen for molecular biology extraction and analysis, while the other was kept at room temperature and promptly brought back to the laboratory for physicochemical analysis.

Soil pH was determined by potentiometric method with 2.5:1 water-soil ratio. Organic carbon content (SOC) was determined by potassium dichromate oxidation-spectrophotometry (Ministry of Environmental Protection, PRC, 2011), and available phosphorus (AP) was determined by molybdenum-antimony anti-color spectrophotometry (Ministry of Agriculture, PRC, 2012). We used potassium chloride solution extraction-spectrophotometry and ultraviolet spectrophotometry to determine ammonium nitrogen (NH4+-N) and the content of Rapid-acting potassium (SK) was determined using the ammonium acetate extraction and flame photometric method.

### 2.3 DNA extraction, amplification, and sequencing

Total genomic DNA was extracted from each soil sample using the TGuide S96 Magnetic Soil/Stool DNA Kit [Tiangen Biotech (Beijing) Co., Ltd.]. The quality and quantity of the extracted DNA were examined using electrophoresis on a 1.8% agarose gel, and DNA concentration and purity were determined with Nano Drop 2000 UV–Vis spectrophotometer (Thermo Scientific, Wilmington, USA). The ITS1 sequence were amplified with primer pairs F: CTTGGTCATTTAGAGGAAGTAA and R: GCTGCGTTCTTCATCGATGC for 25 cycles. The amplification reaction system was as follows: 25 μL of Taq enzyme, 1 μL of 10 mM forward and reverse primers, 3 μL of template DNA (20 ng μL−1), and ddH2O added up to 50 μL. The thermal cycling conditions were: initial denaturation at 95 °C for 5 min; denaturation at 95 °C for 30 s, annealing at 50 °C for 30 s, extension at 72 °C for 40 s, repeated for 25 cycles; final extension at 72 °C for 7 min. PCR amplicons were purified with VAHTSTM DNA Clean Beads (Vazyme, Nanjing, China) and quantified using the Qubit dsDNA HS Assay Kit and Qubit 3.0 Fluorometer (Invitrogen, Thermo Fisher Scientific, Oregon, USA). After the individual quantification step, amplicons were pooled in equal amounts. SMRTbell libraries were prepared from the amplified DNA by SMRTbell Express Template Prep Kit 2.0 according to the manufacturer’s instructions (Pacific Biosciences). Purified SMRTbell libraries from the pooled and barcoded samples were sequenced on a Illumina platform (Beijing Biomarker Technologies Co., Ltd., Beijing, China) using Sequel II binding kit 2.0.

The bioinformatics analysis of this study was performed with the aid of the BMK Cloud.^1^ The raw reads generated from sequencing were filtered and demultiplexed using the SMRT Link software (version 8.0) with the min Passes ≥5 and min Predicted Accuracy ≥0.9, in order to obtain the circular consensus sequencing (CCS) reads. Subsequently, the lima (version 1.7.0) was employed to assign the CCS sequences to the corresponding samples based on their barcodes. CCS reads containing no primers and those reads beyond the length range (1,200–1,650 bp) were discarded through the recognition of forward and reverse primers and quality filtering using the Cut adapt (version 2.7) quality control process (Bolger et al., 2014). UCHIME algorithm (v8.1) was used in detecting and removing chimera sequences to obtain the clean reads (Büchi and Vuilleumier, 2014). Clean reads then were conducted on feature classification to output an ASVs (amplicon sequence variants) by DADA2 (Callahan et al., 2016), and the ASVs counts less than 2 in all samples were filtered. Taxonomy annotation of the ASVs was performed based on the Naive Bayes classifier in QIIME2 (Bolyen et al., 2019) using the SILVA database (Quast et al., 2012) (release 138.1) with a confidence threshold of 70%.

### 2.4 Identification of habitat generalists, specialists, and opportunists of soil fungi

Levins niche width (Equation 6) (Levins, 1968) was calculated using the ‘spaa’ package of R v 4.0.0 (Zhang and Ma, 2014). The frequency of occurrence of each ASV annotate to species level was randomized and rearranged 1,000 times using the replacement method of the ‘EcolUtilis’ package (Salazar, 2019), respectively. Then the zero distribution of the niche width index was calculated for each ASV annotate to species level. Based on the consensus that habitat-generalists owe a wider niche width than the habitat-specialists (Wilson and Hayek, 2015), ASV species were classified into habitat-generalists or specialists based on whether the frequency of observed occurrence exceeded the upper 95% confidence interval or fell below the lower 5% confidence interval, and the others with the observed niche width within in the 5 to 95% confidence interval range were distributed to the habitat-opportunists (Wu et al., 2022).


(6) $$\mathrm{Bi}=\frac{1}{\sum_{j=1}^{r}ij_{2}^{P}}$$

### 2.5 Co-occurrence network and key taxa analysis

The co-occurrence patterns of soil fungi communities among the different forest types were determined using network analysis. Co-occurring networks based on Spearman correlation analysis in this study were conducted using the “Hmisc” package in R program. The co-occurrence patterns of soil fungal communities were studied based on strong correlations (*r* > 0.7) and significant correlations (*p* < 0.05). Network analysis was performed using “igraph” package (Csárdi et al., 2024) and then visualized in Gephi 0.9.

Moreover, the “igraph” package was employed to calculate the within-module connectivity (*Zi*) and between-module connectivity (*Pi*) of the constructed network, aiming to identify key species. Based on within-module connectivity (*Zi*) and among-module connectivity (*Pi*), ASVs were classified into network hubs (*Zi* ≥ 2.5; *Pi* ≥ 0.62), module hubs (*Zi* ≥ 2.5; *Pi* < 0.62), connectors (*Zi* < 2.5; *Pi* ≥ 0.62), and peripherals (*Zi* < 2.5; *Pi* < 0.62) Generally, the other three types of nodes except peripherals were classified as key nodes (Deng et al., 2012), and these nodes played a crucial role in maintaining the stability of the network structure (Hu et al., 2024).

### 2.6 Data analysis

Based on the ASVs abundance matrix, the Shannon-Wiener diversity indices and ASVs species richness for each habitat-specialists were computed. After testing for normality and homogeneity of variances, one-way ANOVA (SPSS 19.0) was used to analyze the significance of soil physicochemical properties, niche width, abundance, and *α*—diversity indices among different afforestation types. Principal Coordinates Analysis (PCoA) function from the ‘vegan’ package was utilized to examine the differences in community structure of each habitat-specialization group among afforestation types. The significance of these structural differences was assessed through a Permanova test (using the adonis function in the vegan package of R software, with permutations = 999 and the Bray-Curtis distance metric). The RDA function was applied to analyze the impacts of tree composition, tree diversity, and soil physicochemical properties on the microbial subgroup community structure. The significance of the RDA model was tested using ANOVA, and the Monte Carlo permutation test was employed to evaluate the significance of the influencing factors of each explanatory variable in the RDA model (via the anova.cca and envfit functions in the vegan package of R software, with permutations = 999). The graph was created using the ggplot2 package in R version 4.2.2.

## 3 Results and analysis

### 3.1 Response characteristics in the abundance of ASVs and niche width of each habitat specialization group to afforestation types

Soil pH and available potassium content of broadleaved forest soil were significantly higher than those of other forest types (*p* < 0.05), indicating that establishing broadleaved forests can more effectively increase soil pH and available potassium content compared to other forest types. The effective phosphorus content in mixed forests and broadleaved forests was higher than that in coniferous forests, but the difference was not significant (*p* > 0.05), suggesting that both mixed forests and broadleaved forests positively contribute to increasing soil effective phosphorus content. However, ammonium nitrogen was significantly higher in coniferous forests and mixed forests compared to broadleaved forests (*p* < 0.05). The organic carbon content showed no significant variation with different forest types (Table 1). The number of sequences obtained from sample sequencing ranged from 35,076 to 61,484, with ASVs varying between 304 and 1,359. Among them, 1,189 ASVs were annotated to the species level. 90 of habitat generalist accounted for 7.57%, 534 of habitat opportunists accounted for 44.91%, and 564 of habitat specialists accounted for 47.43% were identified.

**Table 1:** Results of soil physicochemical properties to afforestation types.

Index stands	Soil organic carbon mg kg^−1^	NH_4_^+^-N mg kg^−1^	Available phosphorus mg kg^−1^	Rapid-acting potassium mg kg^−1^	pH
CF	5.59 ± 3.22a	12.74 ± 4.23a	15.75 ± 13.29a	88.21 ± 50.82b	5.1 ± 0.27b
MF	4.91 ± 2.42a	11.80 ± 2.69a	28.67 ± 19.62a	53.53 ± 95.93b	5.2 ± 0.72b
BF	5.38 ± 0.77a	9.35 ± 5.29b	28.39 ± 23.35a	199.87 ± 22.82a	6.3 ± 0.38a

CF, coniferous forest; BF, broadleaved forest; MF, mixed forest.

**Figure 1:**
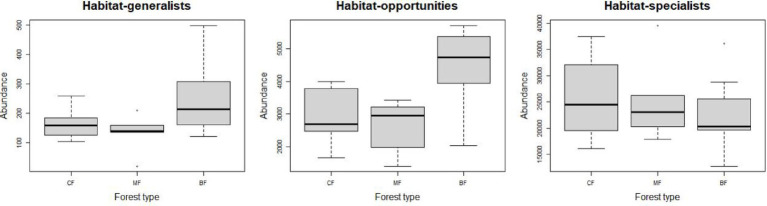
Response of fungal abundance with each habitat specialization group to afforestation types (CF, coniferous forest; MF, mixed forest; BF, broadleaved forest).

**Figure 2:**
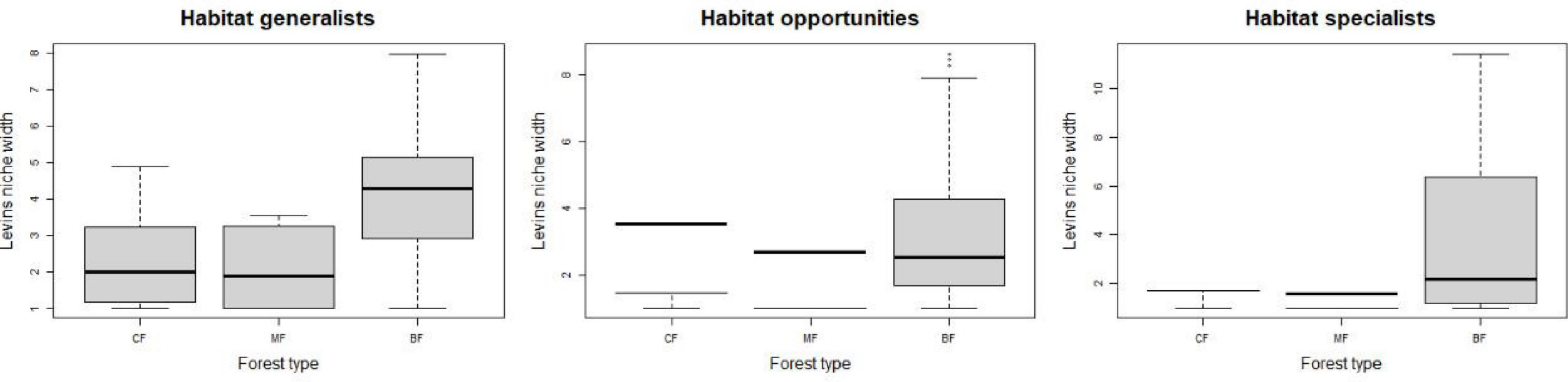
Response of fungal niche breadth among each habitat-specialization group to afforestation types (CF, coniferous forest; MF, mixed forest; BF, broadleaved forest).

The abundance of soil fungi in each type of plantation forests increased with the degree of habitat specialization. In broadleaved forests, the abundance of habitat-generalist and opportunities was higher compared to coniferous and mixed forests, while the abundance of habitat-specialist showed the opposite trend (Figure 1), suggesting a higher degree of habitat specialization in coniferous forest soil environments. No significant difference in niche widths was found among each habitat-specialization group. Among them, habitat generalists showed a significantly higher niche width in broadleaved forests compared to other forest types, while habitat-opportunities and specialists have a higher niche width in coniferous forests (Figure 2).

### 3.2 Response characteristics of α-diversity and community structure of each habitat-specialization group of soil fungi to afforestation types

**Figure 3:**
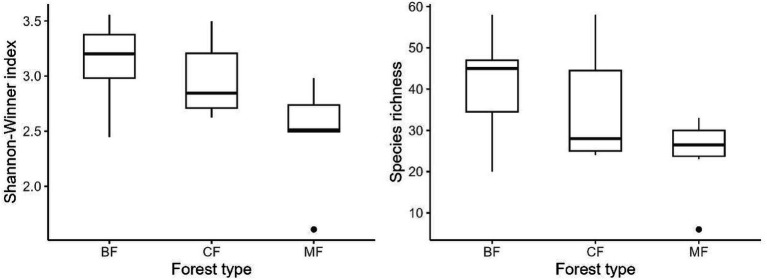
Response of species richness and Shannon-Winner index of habitat generalist to forest stands (CF, coniferous forest; MF, mixed forest; BF, broadleaf forest).

**Figure 4:**
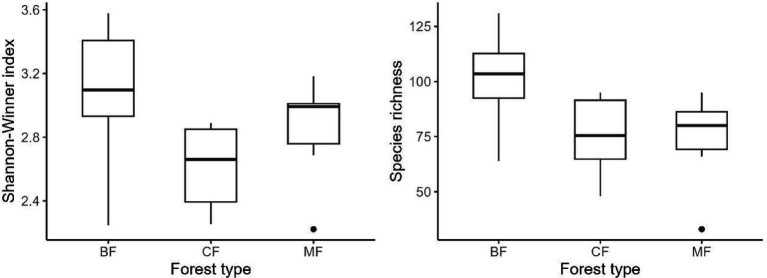
Response of species richness and Shannon-Winner index of habitat opportunists to forest stands (CF, coniferous forest; MF, mixed forest; BF, broadleaved forest).

**Figure 5:**
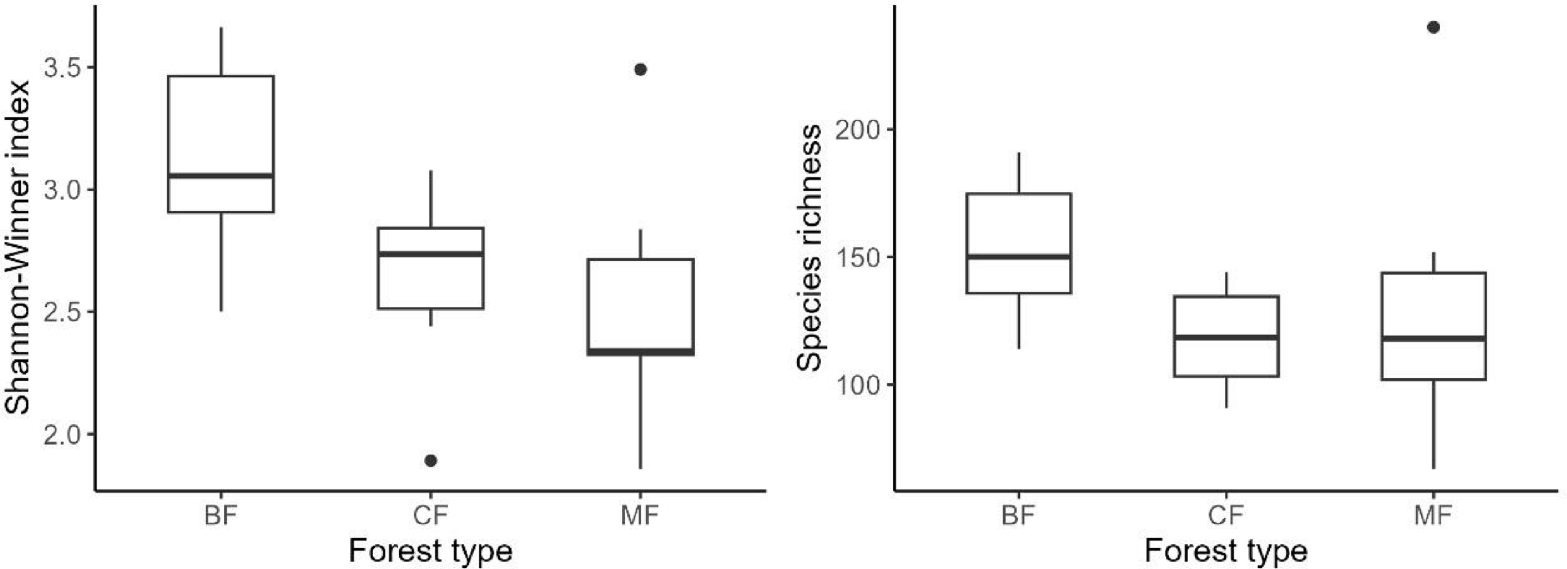
Response of species richness and Shannon-Winner index of habitat specialists to forest stands (CF, coniferous forest; MF, mixed forest; BF, broadleaved forest).

Our research indicated that the Shannon-Winner diversity index and species richness of each habitat-specialization group of soil fungi in broadleaved forests significantly higher than other afforestation types (Figures 3–5). However, the situation differs between coniferous and mixed forests: The diversity indexes were higher in coniferous for habitat-generalists and specialists, while they were higher in mixed forests for habitat-opportunities (Figures 3–5).

**Figure 6:**
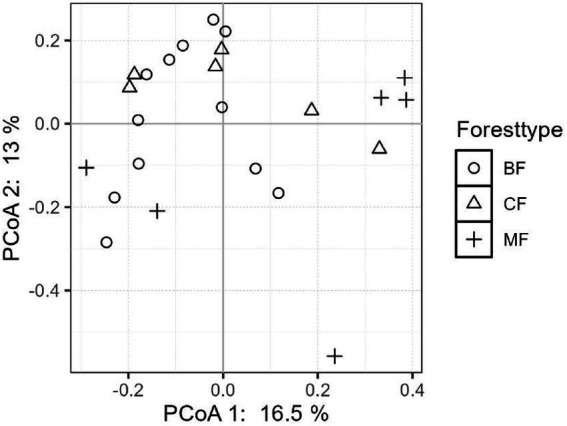
The community structure of habitat-generalists among different forest stands.

**Figure 7:**
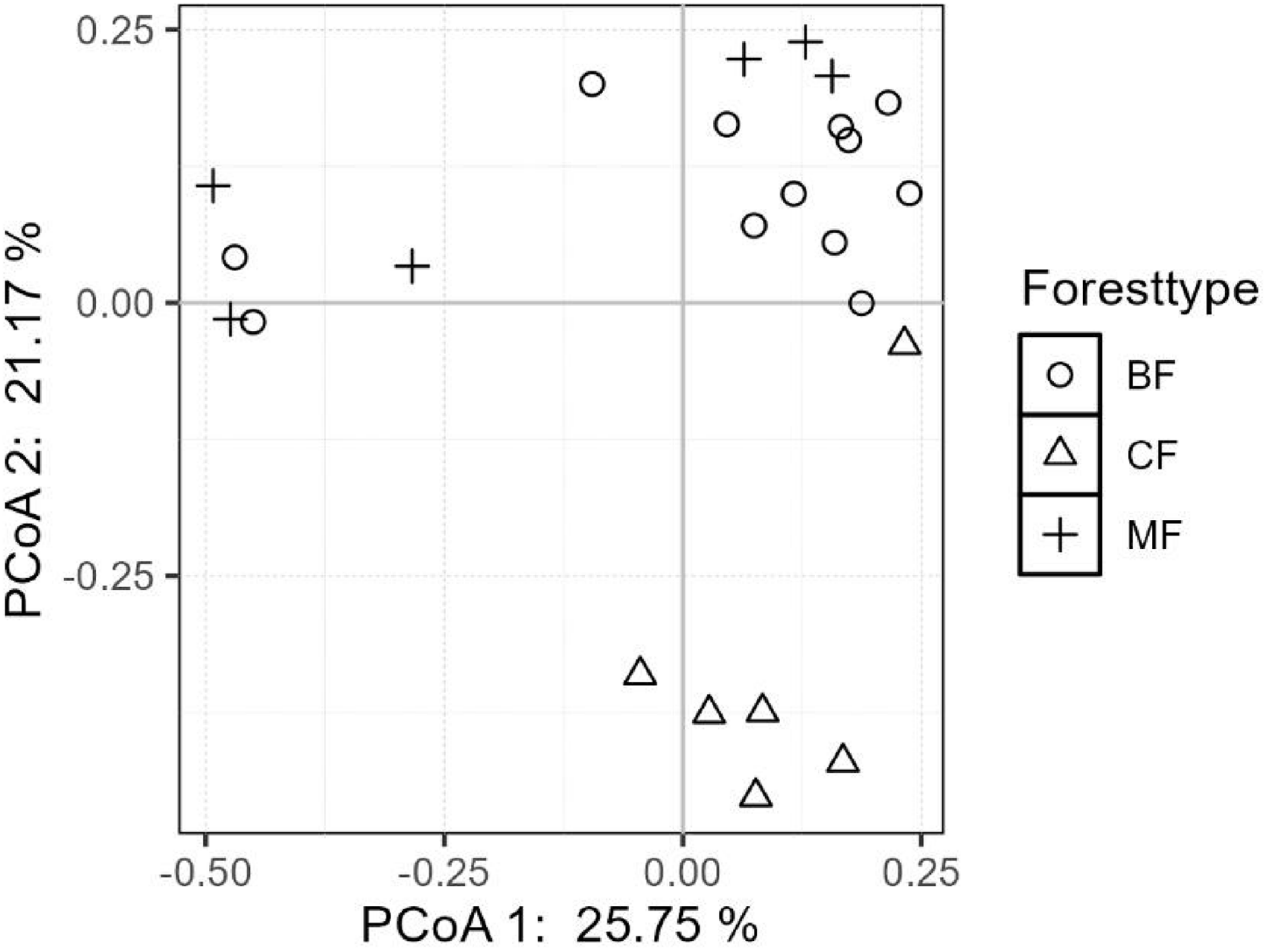
The community structure of habitat-opportunists among different forest stands.

**Figure 8:**
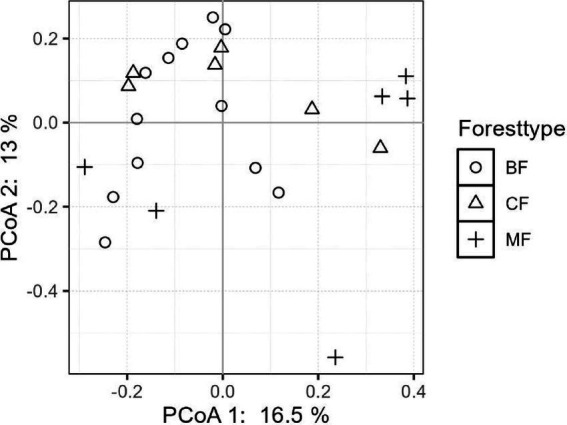
The community structure of habitat-specialists among different forest stands.

The differentiation of community structure among afforestation types increased with the degree of habitat specialization. The community structure of habitat generalist varied greatly within forest type but showed direct mixing between forest types, indicated no significant difference in community structure among afforestation types (Figure 6). In the case of habitat opportunists, the coordinate points of coniferous forests were already clearly separated from those of mixed and broadleaved forests, suggested a clear difference in community structure. The coordinate points of mixed forests were mostly mixed with those of broadleaved forests, suggested no significant difference in the structure of habitat opportunities (Figure 7). For habitat specialists, the coordinate points of different forest stands had distinct aggregation ranges in a two-dimensional graph, indicated a significant difference in community structure among afforestation types (Figure 8).

### 3.3 Environmental driving mechanisms of community structure for each habitat-specialization group among afforestation type

#### 3.3.1 The driving mechanisms of plant-diversity on community structure

**Figure 9:**
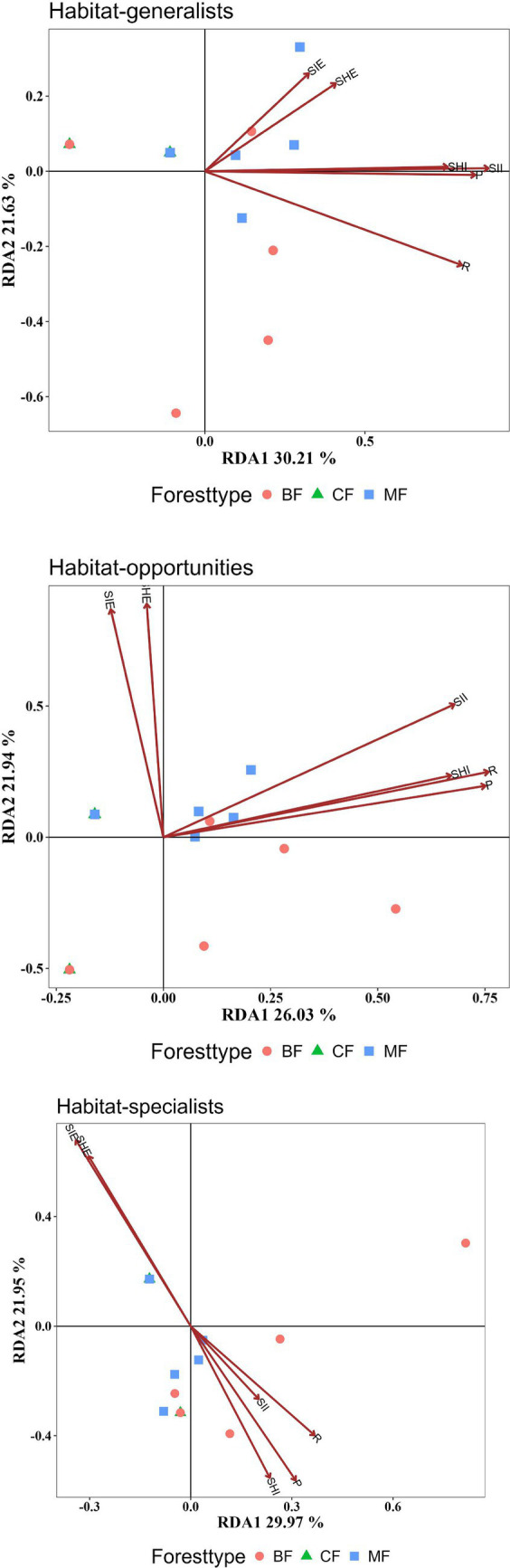
RDA analysis of the impact of plant diversity indices on the structure of each habitat-specialization group (CF, coniferous forest; MF, mixed forest; BF, broadleaved forest; R, Species Richness; SHI, Shannon-Winner Index; SII, Simpson Index; SHE, Shannon Evenness Index; SIE, Simpson Evenness Index; P, Pielou Evenness Index).

Research results indicated the structure of habitat opportunists was more significantly influenced by plant community diversity indices. All diversity indices collectively explain the structure of habitat opportunities at an extremely significant level (*p* < 0.01). In contrast, habitat specialists were not significantly affected by plant diversity indices (Figure 9, Table 2).

**Table 2:** Results of Monte Carlo test for RDA redundancy analysis of plant diversity on each habitat-specialization group.

Model	Habitat-generalists		Habitat-opportunists		Habitat-specialists	
	*F*	*P*	*F*	*P*	*F*	*P*
	0.919	0.701	0.834	0.843	0.791	0.703
Index	*R* ^2^	*P*	*R* ^2^	*P*	*R* ^2^	*P*
R	0.497	**0.001**	0.546	**0.001**	0.162	0.137
SHI	0.390	**0.012**	0.433	**0.005**	0.178	0.105
SII	0.533	**0.001**	0.630	**0.001**	0.059	0.538
SHE	0.159	0.154	0.732	**0.001**	0.236	0.128
SIE	0.128	0.222	0.708	**0.001**	0.286	0.062
P	0.485	**0.001**	0.514	**0.001**	0.207	0.053

Bold indicates *P* < 0.05. R, Species Richness; SHI, Shannon-Winner Index; SII, Simpson Index; SHE, Shannon Evenness Index; SIE, Simpson Evenness Index; P, Pielou Evenness Index.

#### 3.3.2 The driving mechanisms of tree species composition on community structure

**Figure 10:**
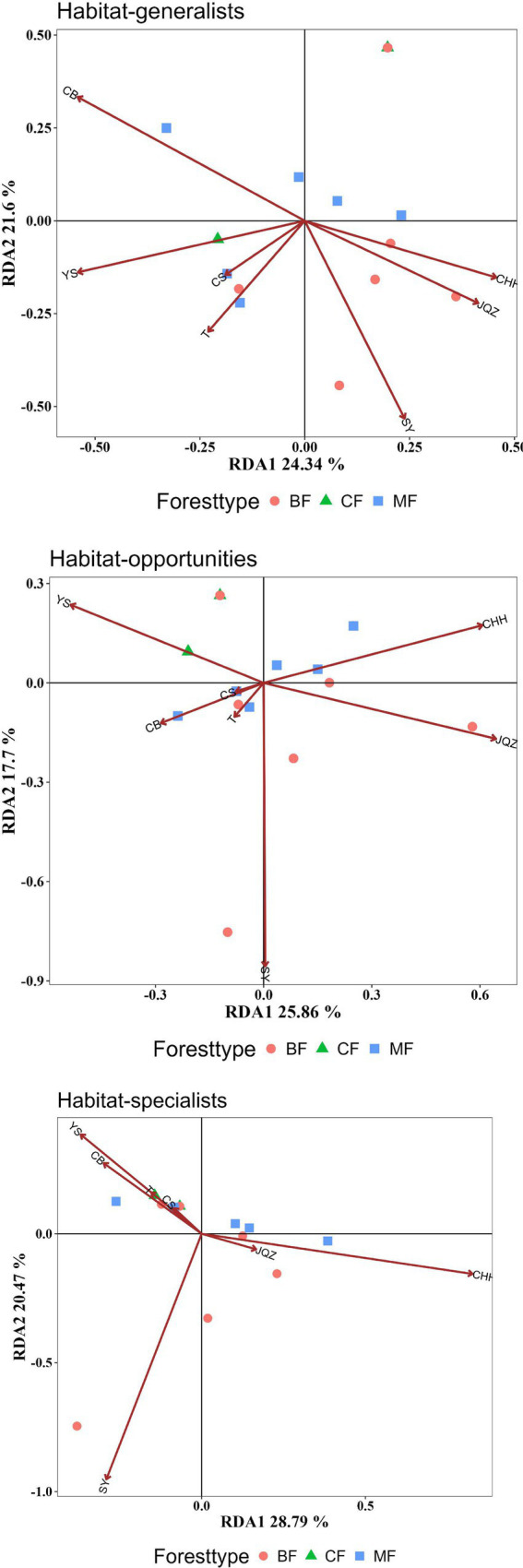
RDA analysis of the impact of plant composition on the structure of each habitat-specialization group (CF, coniferous forest; MF, mixed forest; BF, broadleaved forest; YS: *Pinus tabulaeformis*; CH: *Robinia pseudoacacia*; JQZ: *Diospyros lotus*; SY: *Populus davidiana*; CB: *Platycladus orientalis*; T: *Prunus persica*; CHH: *Pinus densiflora*).

Studies have shown that the differences in the community structure of habitat-specialists between afforestation types were all influenced by the dominant tree species within the communities. The structural differences of habitat generalists among afforestation types were significantly influenced by the importance values of *Populus davidiana* and *Platycladus orientalis*. Habitat-opportunities were significantly affected by the importance values of Pinus tabulaeformis, *Robinia pseudoacacia*, and Populus davidiana. The habitat-specialists were significantly influenced by the importance values of *Robinia pseudoacacia* and *Populus davidiana*. The structural characteristics of each habitat-specialists vary from coniferous forests, mixed forests to broad-leaved forests, showing a positive correlation with the increase of *Populus davidiana* importance values and the decrease of *Pinus tabulaeformis* and *Platycladus orientalis* importance values (Figure 10, Table 3).

**Table 3:** Results of Monte Carlo test for Redundancy Analysis (RDA) of plant composition on each habitat-specialization group.

Model	Habitat-generalists		Habitat-opportunists		Habitat-specialists	
	*F*	*P*	*F*	*P*	*F*	*P*
	1.002	0.481	1.103	0.258	1.000	0.375
Index	*R* ^2^	*P*	*R* ^2^	*P*	*R* ^2^	*P*
*Pinus tabulaeformis*	0.225	0.065	0.291	**0.032**	0.182	0.135
*Robinia pseudoacacia*	0.190	0.101	0.308	**0.014**	0.337	**0.007**
*Diospyros lotus*	0.184	0.148	0.366	0.063	0.015	0.632
*Populus davidiana*	0.272	**0.030**	0.671	**0.003**	0.963	**0.001**
*Platycladus orientalis*	0.339	**0.018**	0.074	0.407	0.100	0.249
*Prunus persica*	0.093	0.378	0.014	0.806	0.031	0.475
*Pinus densiflora*	0.038	0.706	0.005	0.923	0.011	0.667

Bold indicates *P* < 0.05.

#### 3.3.3 The driving mechanisms of soil properties on community structure

**Figure 11:**
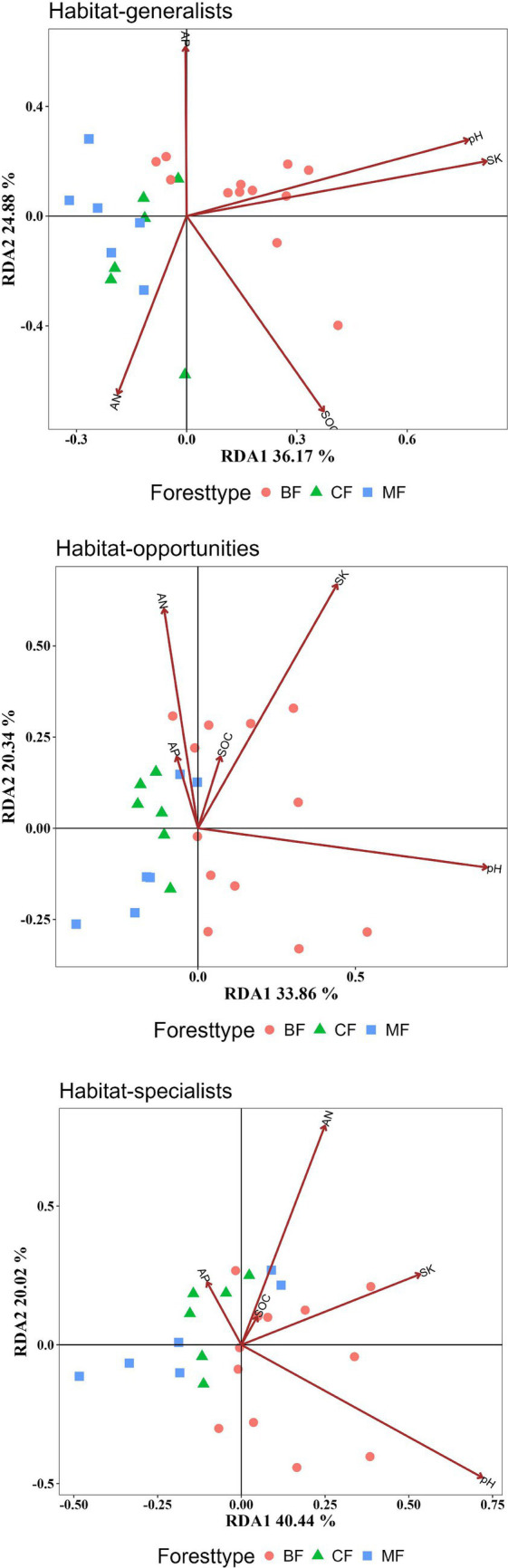
RDA analysis of the impact of soil properties on the structure of each habitat-specialization group (CF, coniferous forest; MF, mixed forest; BF, broadleaved forest).

Habitat-generalists and opportunities tended to be significantly influenced by more soil indicators than specialists. AN and pH significantly affected on the structure of all habitat-specialization groups. Habitat generalist and opportunities of broadleaved forests could be distinctly separated on the RDA1 axis from coniferous and mixed forests, while habitat specialists showed overlapping coordinates across different forest types, indicated that soil properties significantly influence the community structure of generalist and opportunities broadleaved forests. The increase in SK and pH positively correlated with the differences in community structure between broadleaved forests and coniferous forests for each habitat specialization group, whereas AN content was negatively correlated with structural changes in habitat generalists and opportunities and (Figure 11, Table 4).

**Table 4:** Results of Monte Carlo test for Redundancy Analysis (RDA) of soil properties on each habitat-specialization group.

Model	Habitat-generalists		Habitat-opportunists		Habitat-specialists	
	*F*	*P*	*F*	*P*	*F*	*P*
	1.260	**0.027**	1.293	**0.018**	1.265	0.165
Index	*R* ^2^	*P*	*R* ^2^	*P*	*R* ^2^	*P*
SOC	0.022	0.008	0.003	0.669	0.274	0.912
AN	0.161	**0.012**	0.380	**0.021**	0.285	**0.003**
AP	0.219	**0.032**	0.111	0.709	0.507	0.567
SK	0.795	**0.001**	0.227	**0.001**	0.571	0.166
pH	0.717	**0.002**	0.590	**0.001**	0.589	**0.001**

Bold indicates *P* < 0.05. SOC, Soil Organic Carbon; AN, NH_4_^+^-N; AP, Available Phosphorus; SK, Rapid-acting Potassium.

### 3.4 Response characteristics of network structure and key groups of each habitat specialization group to afforestation types

#### 3.4.1 Modularity and topological characteristics of network structure of habitat-specialization groups

Compared to habitat generalists, the network structures of habitat opportunities and habitat specialists were more complex and have higher modularity (Supplementary Figure). Firstly, the connectivity between nodes of habitat specialists was higher, which can be reflected by the higher node degree, hub index, clustering coefficient, eigenvector centrality, and average path length compared to those of habitat opportunities and habitat specialists. These suggested that specialists and opportunities have higher internal connectivity within the network and were more likely to form modular structures. On the contrary, Habitat generalists formed more independent subnetworks, which were structurally more fragile, as evidenced by their higher eccentricity, betweenness centrality, module categories, PageRank values, greater number of components, and shorter average path lengths (see Table 5).

**Table 5:** Comparison of topological parameters of network structures for each habitat specialization group.

Topological parameter	Generalist	Opportunists	Specialist
Weighted degree	4.645	14.915	26.138
Eccentricity	9.656	10.173	9.933
Closeness centrality	0.262	0.226	0.225
Harmonic closeness centrality	0.284	0.256	0.264
Betweenness centrality	1481.117	2291.088	1904.438
Authority	0.000	0.006	0.016
Hub	0.000	0.006	0.016
Modularity class	9.547	11.400	11.711
Page ranks	0.000	0.000	0.001
Component number	0.500	0.362	0.269
Clustering	0.293	0.440	0.529
Triangles	15.421	467.013	1165.649
Eigen centrality	0.001	0.066	0.172

#### 3.4.2 Response of network structures of habitat-specialization groups to forest type

**Figure 12:**
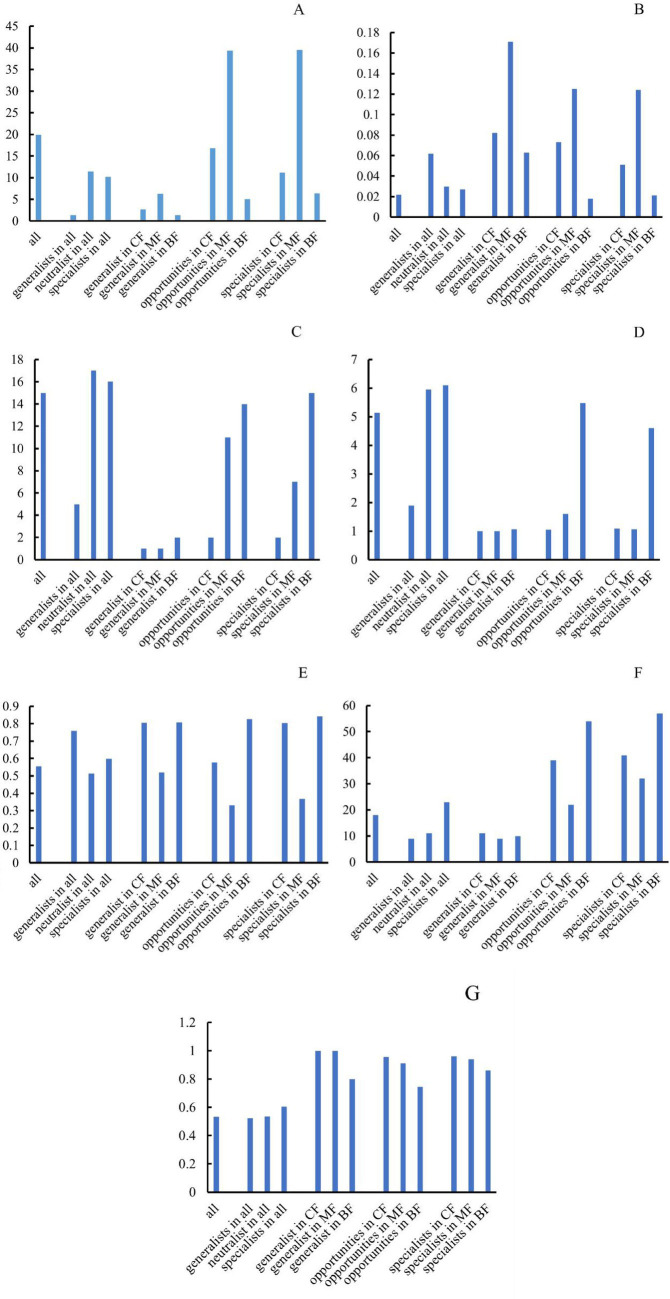
Difference of network topological index among forest stands for each habitat-specialization group (**A**: average weighted degree; **B**: density of graph; **C**: network diameter; **D**: average path length; **E**: modular; **F**: connective component; **G**: average clustering coefficient; CF, coniferous forest; MF, mixed forest; BF, broadleaved forest).

Results indicated that the topological parameters of network structure of each habitat specialization group responded consistently to changes in forest stands. The average degree and graph density were the highest in mixed forests (Figures 12A,B), while modularity and the number of connected components were the lowest (Figures 12E,F). This can be interpreted as the complex soil environment of mixed forests generated more frequent and complex interactions among fungi, lead to higher average degree and graph density. This disrupted the clear boundaries between modules, thereby reducing the modularity index. In broad-leaved forests, the average path length and network diameter were the highest (Figures 12C,D), while the average clustering coefficient were the lowest (Figure 12G), indicating that the fungal network structure in broad-leaved forests is loose with weak node connections. This was also consistent with low graph density and high modularity index in broad-leaved forests.

#### 3.4.3 Key taxa of network structure

**Figure 13:**
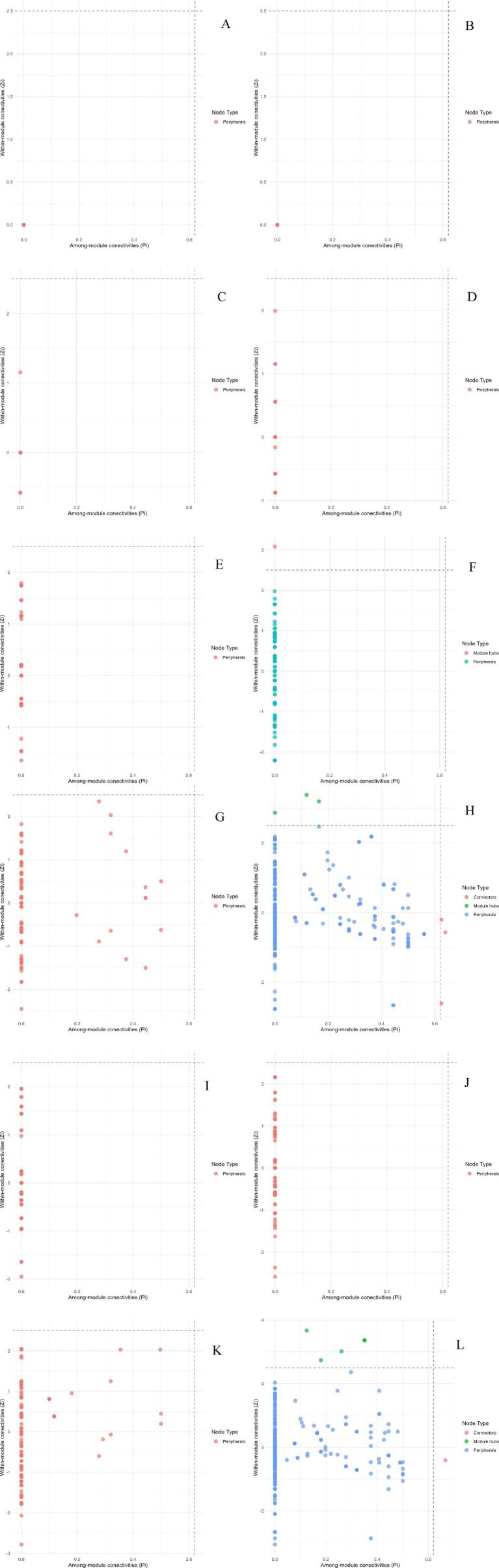
Differences of Zi and Pi of each habitat-specialization group between forest stands (**A**: Generalists of CF; **B**: Generalists of MF; **C**: Generalists of BF; **D**: Generalists of all stands; **E**: Opportunists of CF; **F**: Opportunists of MF; **G**: Opportunists of BF; **H**: Opportunists of all stands; **I**: Specialists of CF; **J**: Specialists of MF; **K**: Specialists of BF; **L**: Specialists of all stands).

Studies have shown that the number of high-value *Zi* and *Pi* increased with the degree of habitat specialization. In the network structure of habitat generalists, only peripheral species were found (Figures 13A–D). For habitat opportunities, 4 species were identified as module hubs, with 1 in mixed forests, and 3 in the overall forest type. Additionally, 3 species were identified as connectors (Figures 13E–H). For habitat specialists, 4 species were identified as module hubs, and 1 was connector (Figures 13I–L). In different types of forest stands, the number of high-value *Zi* and *Pi* showed a trend of increasing from coniferous forests to mixed forests and then to broad-leaved forests. Nodes with Pi>0 were only present in broad-leaved forests, and their numbers were significantly higher in habitat opportunities and habitat specialists than in habitat generalists.

**Figure 14:**
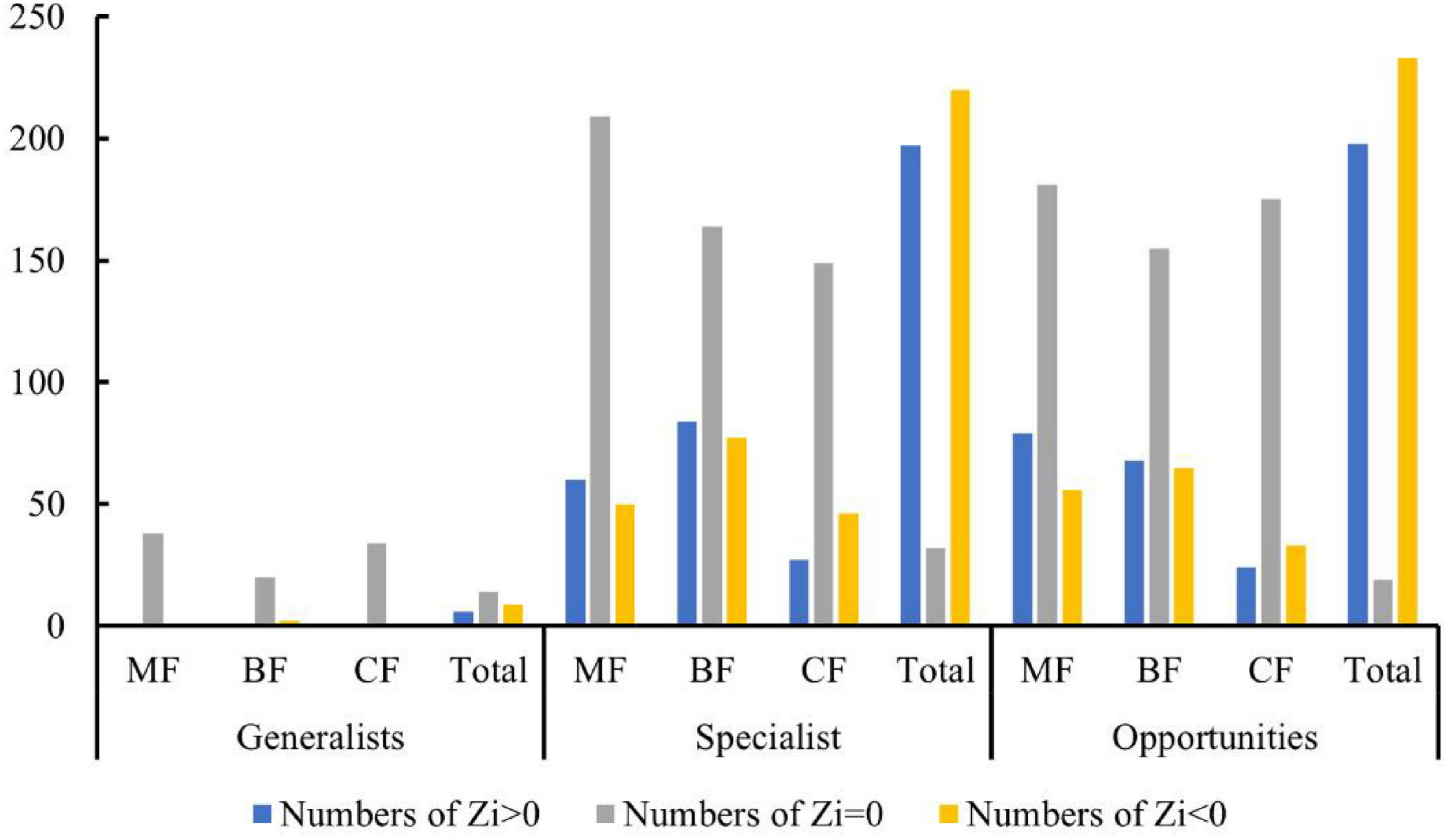
Differences of numbers of positive and negative Zi values among forest types of each habitat-specialization group.

**Figure 15:**
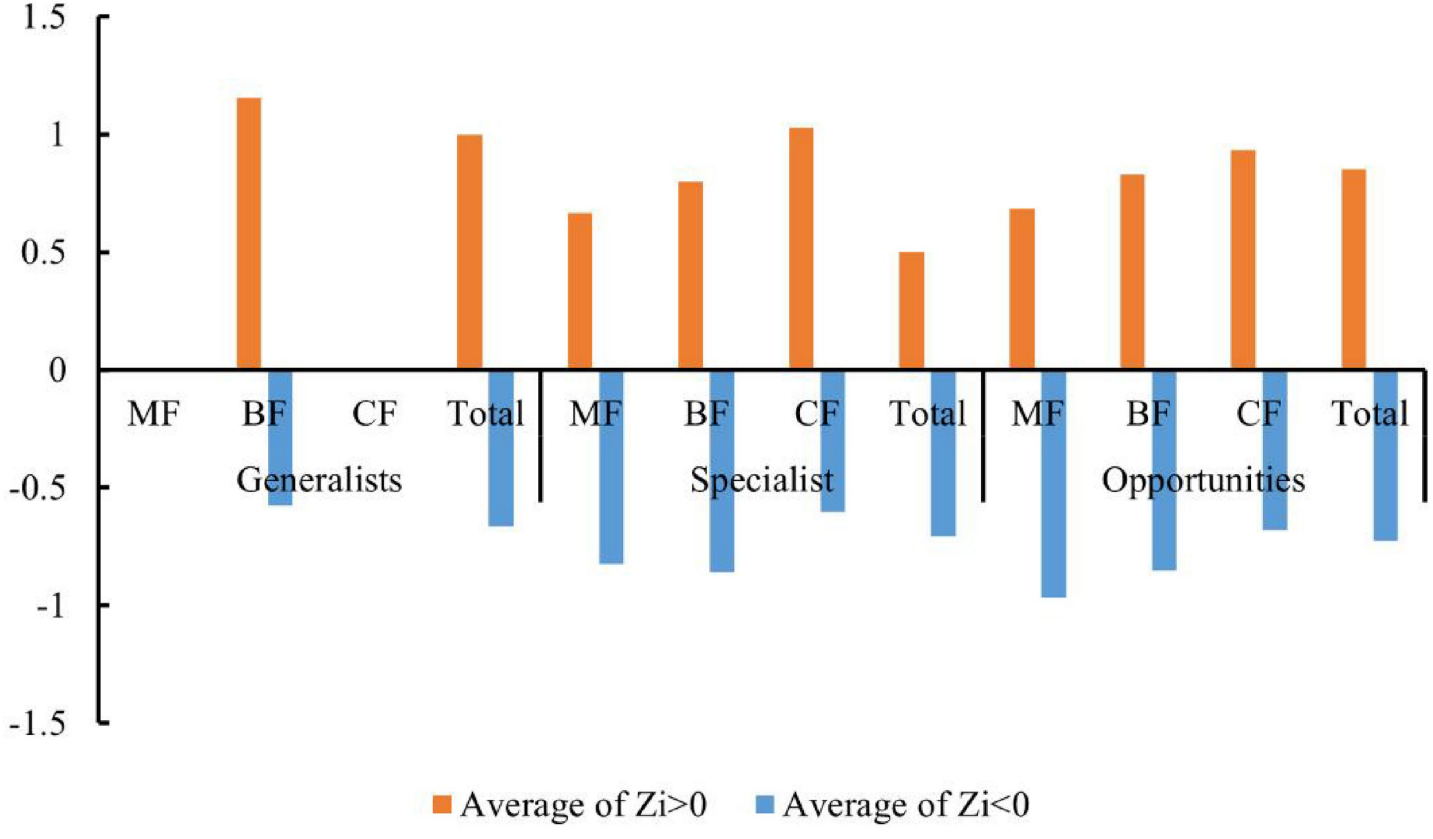
Differences of average positive and negative Zi values among forest types of each habitat-specialization group (CF, coniferous forest; MF, mixed forest; BF, broadleaf forest).

**Figure 16:**
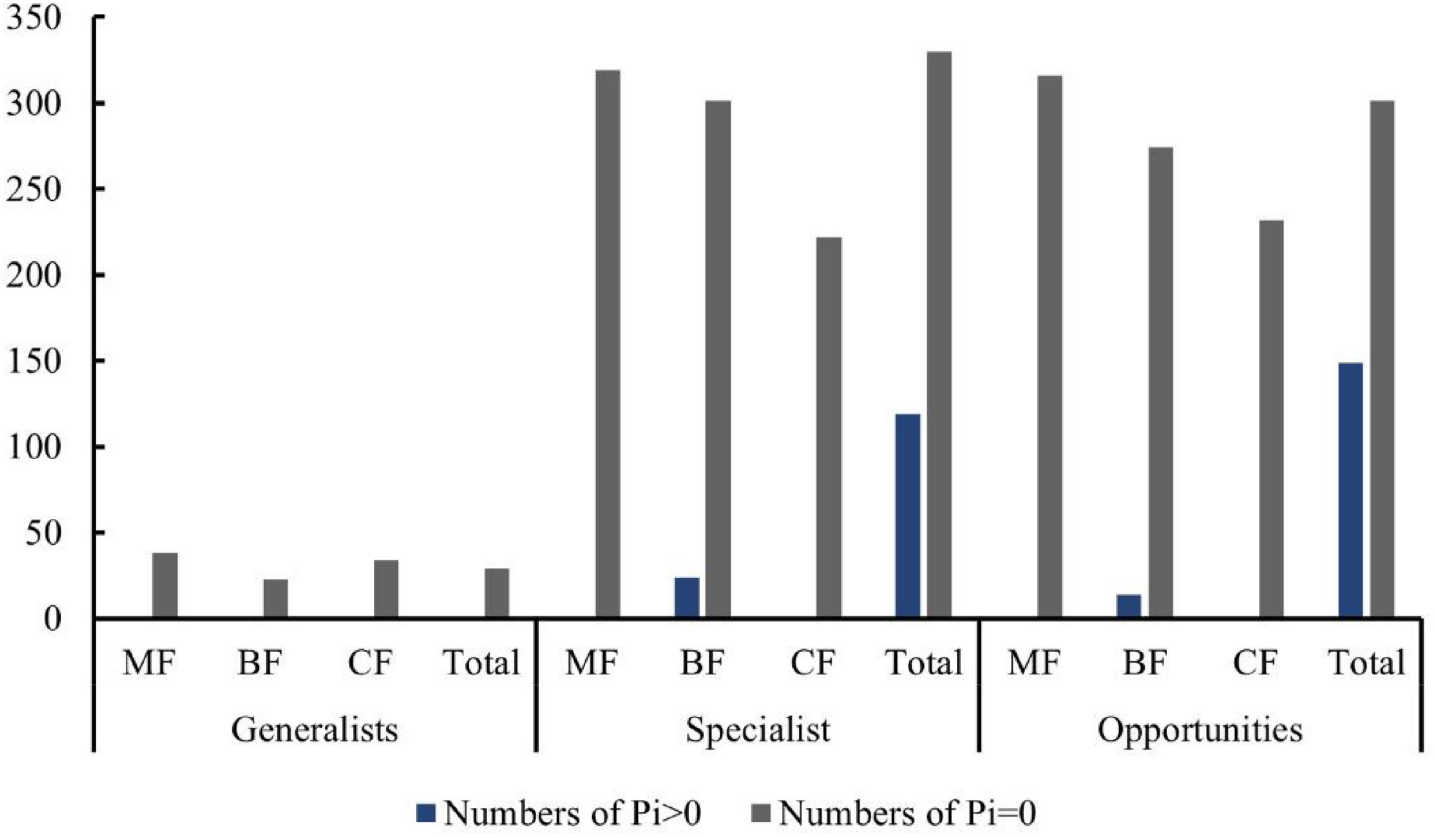
Differences of numbers of positive and 0 Pi values among forest types of each habitat-specialization group (CF, coniferous forest; MF, mixed forest; BF, broadleaf forest).

**Figure 17:**
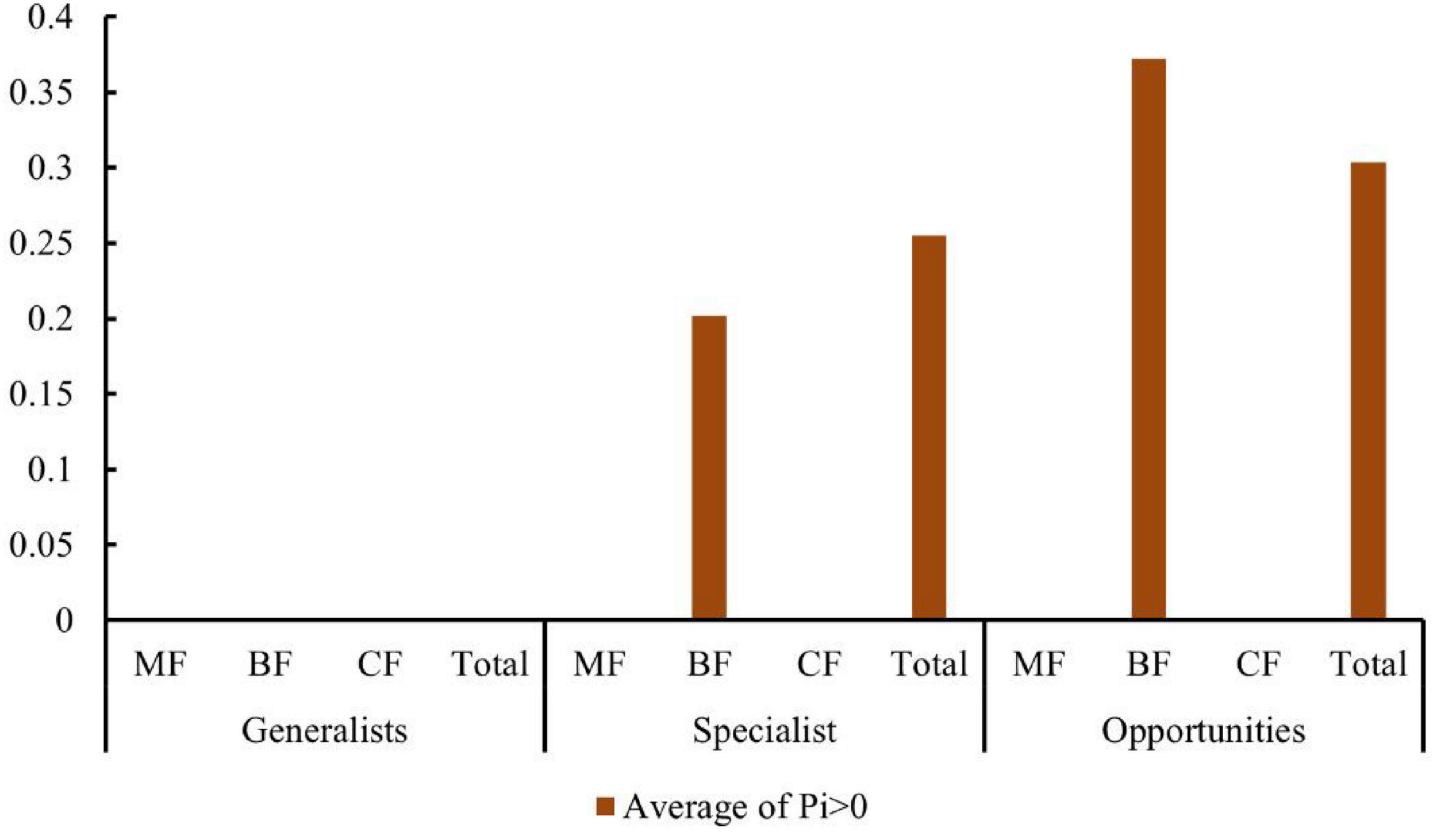
Differences of average Pi values among forest types of each habitat-specialization group (CF, coniferous forest; MF, mixed forest; BF, broadleaf forest).

The distribution characteristics of *Zi* and *Pi* values revealed that planting broad-leaved tree species enhanced the within-module and between-module connections of habitat opportunities and habitat specialists. First of all, the number of negative *Zi* values for habitat opportunities and habitat specialists was greater than the number of positive *Zi* values in coniferous forests, while in mixed forests and broad-leaved forests, the number of positive *Zi* values was greater than the number of negative *Zi* values (Figure 14). In addition, in coniferous forests, the positive mean of *Zi* was greater than the negative mean for habitat opportunities and habitat specialists, while in mixed forests and broad-leaved forests was the opposite trend, with negative mean of *Zi* greater than the positive mean (Figure 15). The connectivity between modules in all habitat specialization groups was poor, with most Pi values being 0. Only a few nodes with positive *Pi* values are present in the broad-leaved forest for habitat opportunities and habitat specialists (Figures 16, 17).

## 4 Discussion

### 4.1 Impact of plantation types on the abundance and α-diversity of soil fungi with different habitat specialization

This study categorized soil fungi based on the confidence interval distribution of niche width, into three major groups: habitat generalists, specialists, and neutrals. Furthermore, the response characteristics of soil fungal community composition and structure, network relationships, and assembly mechanisms to different types of artificial forests were explored along this habitat specialization gradient. Our research results strongly supported the highest heterogeneity of soil environments in broad-leaved forest. Firstly, the abundance of habitat-generalists was the highest in broad-leaved forests, this group demonstrated a higher tolerance to environmental changes, effectively utilizes diverse resources, and thrives across various habitats (Wan et al., 2021; Jia et al., 2018; Xu et al., 2021). Secondly, the niche breadth of habitat generalists was higher in broad-leaved forests than in other forest types, and it is higher than that of habitat specialist species in all forest types. Finally, the diversity of similarly habitat-specialized groups showed a trend of broad-leaved forests > mixed forests > coniferous forests, indicating that the soil environment of broad-leaved forests is suitable for the coexistence of more fungal species, which verifies its high environmental heterogeneity. In addition, the study also found the number and abundance of specialist and opportunities significantly higher than generalists, indicated that most fungal members had a limited distribution range, while only a small portion were highly distributed in space and time (Sriswasdi et al., 2017; Delgado-Baquerizo et al., 2018). This finding aligned with the conclusions of Li et al. (2025), and could also be expected that the different afforestation types studied exert strong selection pressure on fungi (Logares et al., 2013). The abundance of habitat-specialists was the highest in coniferous forests, indicating that the soil environment in coniferous forests has the highest degree of specialization and exerts the strongest filtering effect on fungal community assembly. The reason was why the specialists rely heavily on specific resource conditions, showing stronger competitiveness in more stable environments and playing key hub and regulatory roles in maintaining community structural stability (Liu et al., 2023).

### 4.2 Impact of plantation types on the β-diversity and environmental driving mechanisms of soil fungi with different habitat specialization

Soil fungal community was influenced by both deterministic and stochastic processes, with the former mainly including interspecific interactions such as coexistence, competition, and exclusion caused by environmental selection, and the latter mainly including random diffusion effects (Peng et al., 2021; Wan et al., 2021). Both processes play an equally important role in driving community assembly of habitat-specialized clades (Büchi and Vuilleumier, 2014). This study showed that the structural turnover rate of habitat-specialists was higher than generalists and opportunities, consistent with the view that the community assembly of habitat specialists was mainly driven by deterministic processes because their narrow environmental tolerance range, making them easily replaced by other species (Lindh et al., 2016), and they were less affected by random diffusion (Liao et al., 2016). The distribution pattern of habitat generalists was more neutral, attributable to their wider ecological adaptability, reduced impact from environmental protection, and increased influence from stochastic factors (FAO, 2022). In coniferous forests, the homogeneity of structures of habitat-specialists was higher than generalists, whereas in broadleaved forests, there was no significant difference in the heterogeneity of each habitat-specialization group structures, confirmed the specialized soil environments in coniferous forests and high heterogeneity of soil environments in broad-leaved forests. These differences in the structures of habitat specialists among afforestation types reflected that the balance between deterministic and stochastic processes can be regulated by environmental factors (Zhang et al., 2019). RDA analysis indicated that changes in community dominant tree species significantly affect the community structure of each habitat-specialized group, consistent with the view of Urbanová et al. (2015). Porazinska et al. (2018) also proposed that particular plant species, rather than plant diversity, more effectively influence the structure of soil microbial communities. Changes in soil properties resulted in significant differences in the community structure of generalist and opportunities between broadleaved forests and other forest types, while little impacted on specialists. This could be attributed to the high environmental heterogeneity of broadleaved forest soils, which weakens the response strength of specialists to environmental changes and reduces their susceptibility to deterministic processes (Mo et al., 2021; Gad et al., 2020). Among these, SK and pH were identified positively correlated to the difference of structure between broadleaved forest and other forests for each habitat specialization group and AN was proved to be negatively correlated to that for generalists and opportunities (Barcenas-Moreno et al., 2016; Dawud et al., 2016).

### 4.3 Impact of plantation types on network structural features of soil fungi with different habitat specialization

Co-occurrence network analysis served as a crucial basis for determining community assembly mechanisms (Xue et al., 2017; Zhou et al., 2021), it also provided powerful tools for interpreting microbial interactions, identifying key species, and calculating topological features (Guan et al., 2021). Relevant studies indicated the key taxa an important role in maintaining network structure, primarily existing as module hubs, and their disappearance may disrupt the network structure (Sun et al., 2021). Our study concluded that the habitat-specialists were more crucial in preserving network structure and stability than habitat generalists and opportunities. Firstly, habitat specialists exhibited higher node degree, centrality and modularity in network topology compared to habitat generalists and opportunities, aligning with the finding of Mo et al.’s (2021). Secondly, the number of key taxa of network in habitat-specialists was notably higher compared to habitat generalist and opportunities. The Key taxa in habitat-specialists mainly served as module hubs than connectors, underscoring their critical role in maintaining network structure, aligning with the findings of Li et al. (2024). In addition, habitat specialists exhibited the highest node degree and graph density and the lowest modularity in mixed forests, whereas the showed higher modularity indices in coniferous and broad-leaved forests. This could be explained as the connectivity of soil fungi in mixed forests increased interactions among species, breaking down module boundaries and thus lowering the modularity index. In mixed and broad-leaved forests, the positive interactions between habitat specialists and opportunities exceeded the negative ones, with opposite in coniferous forests. This suggested that coexistence and symbiosis dominate among habitat specialists and opportunities in mixed and broad-leaved forests, while competition and exclusion prevail in coniferous forests (Liu et al., 2024).

## 5 Conclusion

From the perspective of habitat specialization, this study systematically revealed the impact characteristics of different afforestation types in limestone mountainous areas on soil fungal diversity, community structure, and co-occurrence networks. It concluded that broad-leaved forests can enhance the diversity of habitat-specialists and strengthen the hub function of their network by increasing soil environmental heterogeneity. This research provided a clear theoretical basis and strategic reference for optimizing underground biodiversity and enhancing ecosystem stability through tree species configuration in the ecological restoration of plantations.

## Data Availability

The datasets presented in this study can be found in online repositories. The names of the repository/repositories and accession number(s) can be found in the article/Supplementary material.

## Footnotes

^1^ http://www.biocloud.net/
