# Supplementary material for: Response of soil fungal community structure and co-occurrence network features on plantations of limestone mountains along habitat specialization gradient
Source: Front Microbiol. 2025 Nov 3;16:1691167. doi: 10.3389/fmicb.2025.1691167 (PMC12620503; doi:10.3389/fmicb.2025.1691167)
Supplement: Supplementary file 1 [file Data_Sheet_1.PDF]

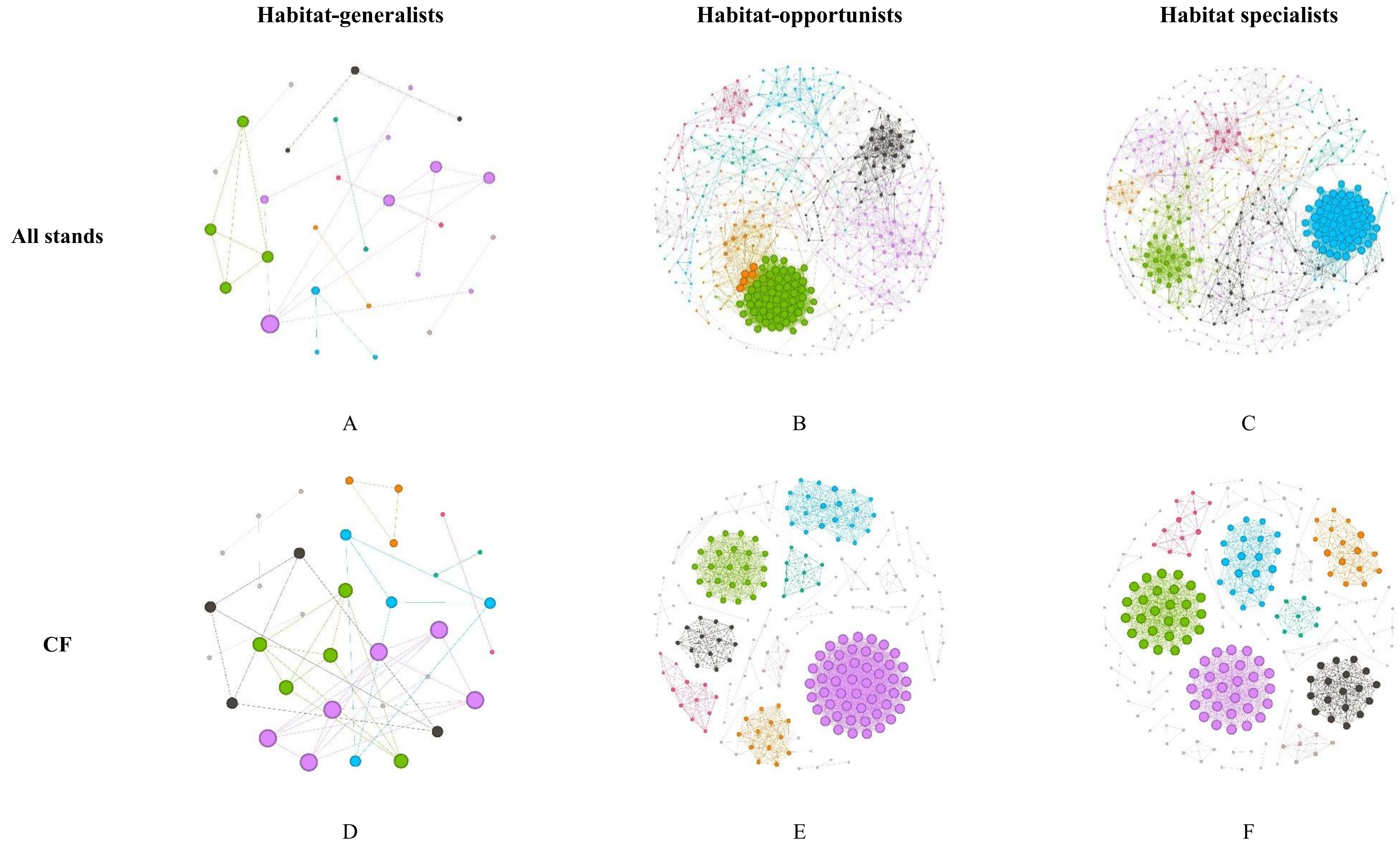

Habitat-generalists
Habitat-opportunists
Habitat specialists
All stands
A
B
C
CF
D
E
F

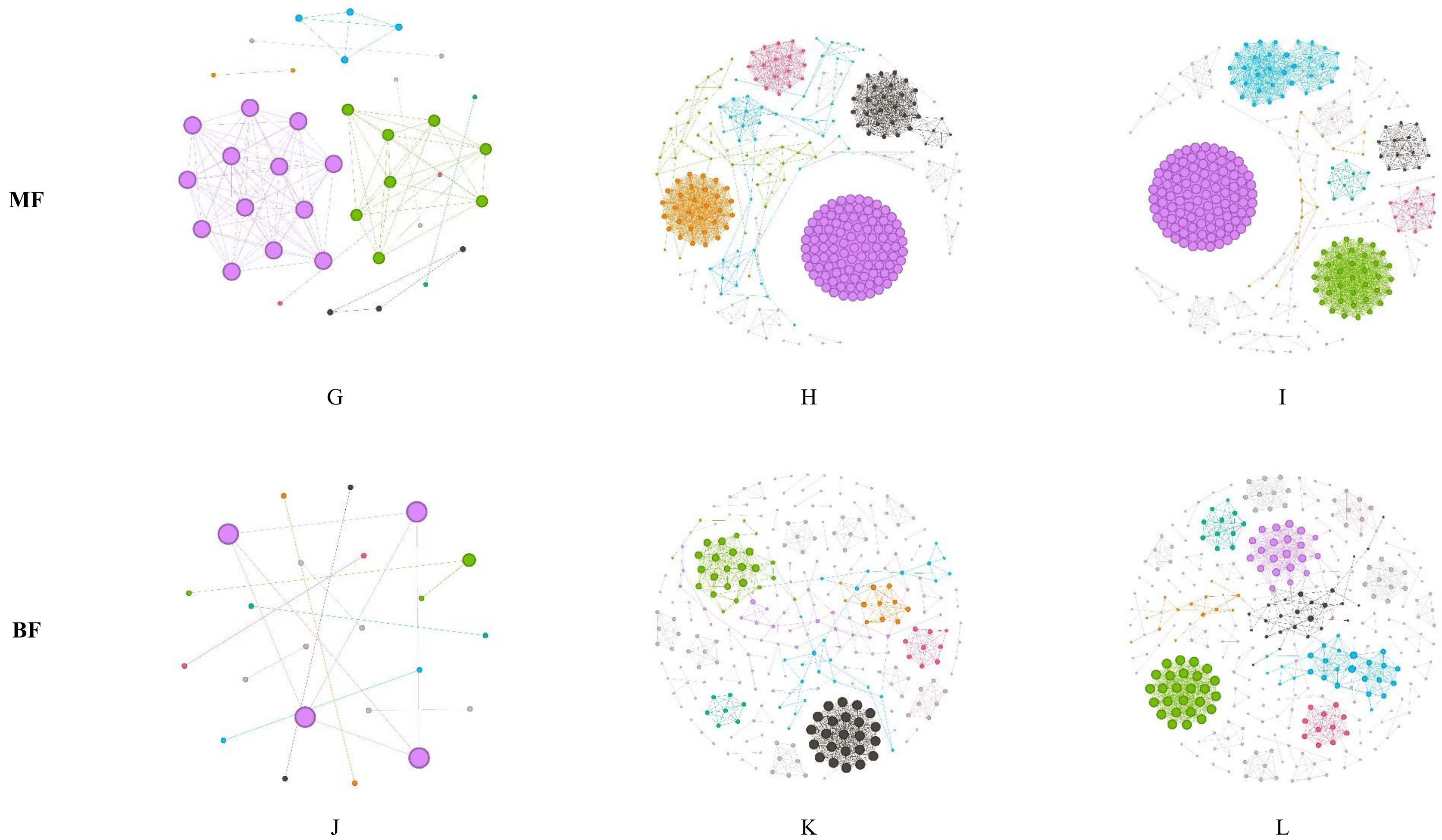

Appendix: Co-occurrence network structure of habitat-specialization groups in different afforestation types and all stands (CF: coniferous forest; MF: mixed forest; BF: broadleaf forest)
